# G-protein coupled receptor solubilization and purification for biophysical analysis and functional studies, in the total absence of detergent

**DOI:** 10.1042/BSR20140171

**Published:** 2015-04-16

**Authors:** Mohammed Jamshad, Jack Charlton, Yu-Pin Lin, Sarah J. Routledge, Zharain Bawa, Timothy J. Knowles, Michael Overduin, Niek Dekker, Tim R. Dafforn, Roslyn M. Bill, David R. Poyner, Mark Wheatley

**Affiliations:** *School of Biosciences, University of Birmingham, Edgbaston, Birmingham, B15 2TT, U.K.; †School of Life and Health Sciences, Aston University, Birmingham B4 7ET, U.K.; ‡School of Cancer Studies, University of Birmingham, Edgbaston, Birmingham, B15 2TT, U.K.; §Discovery Sciences, AstraZeneca R&D Mölndal, 43183 Mölndal, Sweden.

**Keywords:** adenosine receptor, detergent-free, G-protein coupled receptor (GPCR), protein thermostability, structure, A_2A_R, adenosine A_2A_ receptor, A_2A_R–SMALP, SMALP-solubilized A_2A_R, A_2A_R–DDM, DDM-solubilized A_2A_R, AUC, analytical ultracentrifugation, cv, column volumes, DDM, *n*-dodecyl-*β*-D-maltopyranoside, FTICR, Fourier-transform ion cyclotron resonance, GPCR, G-protein coupled receptor, HEK, human embryonic kidney, MSP, membrane scaffold protein, NECA, 5'-(*N*-ethylcarboxamido)adenosine, NTA, nitrilotriacetate, PTH1R, parathyroid hormone 1 receptor, SMA, styrene maleic acid, SMALP, SMA lipid particle, XAC, xanthine analogue congener

## Abstract

G-protein coupled receptors (GPCRs) constitute the largest class of membrane proteins and are a major drug target. A serious obstacle to studying GPCR structure/function characteristics is the requirement to extract the receptors from their native environment in the plasma membrane, coupled with the inherent instability of GPCRs in the detergents required for their solubilization. In the present study, we report the first solubilization and purification of a functional GPCR [human adenosine A_2A_ receptor (A_2A_R)], in the total absence of detergent at any stage, by exploiting spontaneous encapsulation by styrene maleic acid (SMA) co-polymer direct from the membrane into a nanoscale SMA lipid particle (SMALP). Furthermore, the A_2A_R–SMALP, generated from yeast (*Pichia pastoris*) or mammalian cells, exhibited increased thermostability (∼5°C) compared with detergent [DDM (*n*-dodecyl-*β*-D-maltopyranoside)]-solubilized A_2A_R controls. The A_2A_R–SMALP was also stable when stored for prolonged periods at 4°C and was resistant to multiple freeze-thaw cycles, in marked contrast with the detergent-solubilized receptor. These properties establish the potential for using GPCR–SMALP in receptor-based drug discovery assays. Moreover, in contrast with nanodiscs stabilized by scaffold proteins, the non-proteinaceous nature of the SMA polymer allowed unobscured biophysical characterization of the embedded receptor. Consequently, CD spectroscopy was used to relate changes in secondary structure to loss of ligand binding ([^3^H]ZM241385) capability. SMALP-solubilization of GPCRs, retaining the annular lipid environment, will enable a wide range of therapeutic targets to be prepared in native-like state to aid drug discovery and understanding of GPCR molecular mechanisms.

## Abstract 2

It is universally acknowledged that exposing cell-surface receptors to detergent is detrimental. We have used a polymer to extract the receptor and surrounding lipid as a nanoparticle that provides a novel solution compatible with purification and receptor-based drug discovery assays.

## INTRODUCTION

G-protein coupled receptors (GPCRs) constitute the largest class of membrane proteins in the human genome with >800 unique receptors. They are activated by an extremely diverse range of stimuli which differ with respect to their physico-chemical properties and size; ranging from photons, through small biogenic amines to peptides and glycoproteins [1]. GPCRs share a common protein architecture comprising seven transmembrane helices and are represented in the genome of organisms from viruses and slime moulds to plants and humans. Consequently, these receptors are central to cell signalling and as such are important to the pharmaceutical industry as therapeutic targets in drug discovery programs. Indeed, 40%–50% of clinically-prescribed drugs and ∼25% of the top selling drugs target this protein family [2].

A pre-requisite to a detailed understanding of the molecular mechanisms underlying ligand binding, receptor activation and intracellular signalling is the ability to study the receptor protein in isolation while preserving the GPCR protein-fold and pharmacology. Extracting GPCRs from the cell membrane to produce a solubilized preparation for studying receptor structure and function has universally involved the use of a class of surfactants often referred to as detergents. These detergents not only disrupt the cell membrane but also simultaneously substitute for the native lipid in close association with the membrane protein (annular lipid). Unfortunately, inserting a protein into a detergent micelle provides only a poor approximation of the cell membrane bilayer and the native annular lipid. Indeed, it is thought that delipidation may be the most common cause of membrane protein inactivation during solubilization [3]. Once solubilized by a detergent, all subsequent studies require the continued presence of detergent to prevent receptor aggregation, despite the problems this causes with respect to receptor stability. This inherent instability of GPCRs in detergent micelles was the motivation for the development a new class of detergent, maltose-neopentyl glycol amphiphiles, designed specifically to improve GPCR stability during solubilization and crystallization studies [4]. An alternative strategy is to exchange the detergent used to solubilize GPCRs [often *n*-dodecyl-*β*-D-maltopyranoside (DDM)] with a non-conventional surfactant, such as amphipathic polymers (amphipols) or fluorinated surfactants [5,6]. The use of these non-conventional surfactants does not obviate the use of classical detergents however, as amphipols and fluorinated surfactants are usually incapable of solubilizing proteins directly from the plasma membrane [3]. Using an alternative strategy, an engineered β-sheet peptide was used to stabilize detergent-solubilized membrane proteins, including the glucagon receptor [7]. Another approach, pioneered by the Sligar laboratory [8] and subsequently utilized by other groups, is to encapsulate detergent-solubilized receptors into nanodiscs [9–11]. Nanodiscs are nanometre-scale planar discs of lipid bilayer stabilized by encircling membrane scaffold protein (MSP), usually a modified form of human high-density lipoprotein apoA-1 [12,13]. This encapsulation of GPCRs into nanodiscs is usually performed using receptor that was previously purified in detergent. However, the parathyroid hormone 1 receptor (PTH1R) was recently purified in nanodiscs following initial solubilization by the detergent DDM, specifically to reduce the time the PTH1R was exposed to detergent [14]. Nevertheless, a general limitation of nanodiscs is that interference from the obligatory stabilizing scaffolding protein prevents or perturbs the downstream application of many biophysical approaches to study the encapsulated protein of interest.

There has been an absolute requirement for detergent to solubilize GPCRs despite the universal acknowledgement that exposing GPCRs to detergent perturbs and destabilizes the receptor. Although significant progress has been made to reduce the detrimental impact of detergent by employing non-conventional surfactants or nanodiscs, the ideal situation would be to generate GPCRs in aqueous solution in a native-like state, complete with annular lipid, but without any exposure to detergent. In the present study, we report the first solubilization and purification of a GPCR, the human adenosine A_2A_ receptor (A_2A_R), without the use of detergent at any stage by exploiting spontaneous encapsulation of a GPCR direct from a membrane into a nanoscale styrene maleic acid (SMA) lipid particle (SMALP). This generates a nano-section of the native membrane excised and stabilized by a ring of SMA polymer [15]. In addition, we demonstrate the utility of our approach by applying SMALP-solubilization to two applications fundamentally important to GPCR research: (i) purification of milligram amounts of functional GPCR from a commonly employed overexpression system (yeast) and (ii) solubilization of GPCR from transfected mammalian cells in culture, with retention of the annular native membrane environment, for downstream applications, such as drug discovery assays.

## EXPERIMENTAL

### Materials

[^3^H]ZM241385 (specific activity 50.0 Ci/mmol) was purchased from American Radiolabelled Chemicals. ZM241385 {4-(2-[7-amino-2-(2-furyl)[1,2,4]triazolo[2,3-a][1,3,5]triazin-5-yl amino]ethyl)phenol} was purchased from Tocris, xanthine analogue congener (XAC), 5'-(*N*-ethylcarboxamido)adenosine (NECA) and theophylline from Sigma. Cell culture media, buffers and supplements were purchased from Invitrogen. Restriction enzymes were obtained from NEB.

### Expression of A_2A_R in *P. pastoris*

General protocols for heterologous expression of A_2A_R in *P. pastoris* were as described in the *Pichia* Fermentation Process Guidelines (Invitrogen) or as described previously [16,17]. The human A_2A_R was expressed with an N-terminal His_10_-tag in the pPICZαA expression plasmid and incorporated an Asn154Gln mutation to preclude hyperglycosylation [18]. The A_2A_R–pPICZαA expression plasmid was linearized, purified and used to transform *P. pastoris strain* X-33 by electroporation. Colonies were selected on yeast extract peptone dextrose sorbitol plates containing 0.1 mg/ml Zeocin and then used for receptor expression screening. Small-scale screening and protein detection was carried out as described previously [16] with the following modifications; the induction temperature was lowered to 22°C and the A_2A_R antagonist theophylline (10 mM) was added at the point of induction [18]. For large-scale expression of A_2A_R, frozen glycerol stock culture was used to inoculate 50 ml of buffered complex glycerol medium (1% yeast extract, 2% peptone, 1.34% yeast nitrogen base without amino acids, 0.00004% biotin, 1% glycerol, 0.1 M phosphate buffer, pH 6) containing 100 μg/ml Zeocin. The cells were grown at 30°C and 220 rpm overnight to yield an optical density at 600 nm (*D*_600_) of 2–6. A 50 ml aliquot of this culture was used to inoculate a 1 l of fermenter (Applikon) containing basal salts medium (BSM) plus *Pichia* trace minerals 1 (PTM_1_) trace salts to a starting *D*_600_ of 0.3. The fermentation run in ‘fed-batch’ mode at 30°C and pH 5.0 was maintained using undiluted (28%) ammonium hydroxide. Dissolved oxygen (DO) was maintained above 20% saturation by adjusting agitation rate and pure oxygen supply.

When the initial glycerol (40 g/l) in batch phase was depleted, as indicated by a spike in DO reading, a 50% (w/v) glycerol solution containing 1.2% (v/v) PTM_1_ was introduced at a feed rate of 30 ml/h for 4 h. Glycerol feed was terminated followed by a 3 h starvation phase to achieve complete glycerol consumption. During the final hour of starvation, the temperature was reduced from 30°C to 22°C and allowed to stabilize. Theophylline (10 mM) was then added to the culture to increase A_2A_R stabilization during expression. The cells were induced with 100% methanol containing 1.2% (v/v) PTM_1_ at an initial feed rate of 1.92 ml/h for 17 h to allow culture adaptation to methanol. When a steady DO rate and fast DO spike time was obtained, indicative of adaptation to methanol utilization, the feed rate was increased to 3.96 ml/h for the remainder of the fermentation. The entire methanol ‘fed-batch’ phase lasted approximately 40 h with a total of ∼125 ml of methanol fed per litre of initial volume. The cells were then harvested by centrifugation at 6000 ***g*** in a Beckman JLA-8.1 rotor and washed once with 10 mM Tris/HCl buffer, pH 8.

### Yeast Membrane preparation

Yeast cells expressing A_2A_R were disrupted as described previously [16]. The membranes were resuspended to 80 mg/ml in 50 mM Tris/HCl, 10% glycerol, 500 mM NaCl, pH 8 and used immediately for SMA solubilization or snap-frozen and stored at −80°C until further use.

### HEK293T cell culture and transfection

Human embryonic kidney (HEK)293T cells were routinely cultured in Dulbecco's modified Eagles medium (DMEM) containing L-glutamine (2 mM), D-glucose (4500 mg/l) and sodium pyruvate (1mM) supplemented with 10% (v/v) fetal calf serum (FCS) in humidified 5% (v/v) CO_2_ in air at 37°C. Transfection was essentially as described previously [19]. Cells were seeded at a density of ∼5 × 10^5^ cells/100-mm dish and transfected after 48 h using a mixture of 5 μg of DNA, 60 μl of polyethyleneimine (10 mM) and 1 ml of 5% glucose solution, which was incubated for 30 min at room temperature before addition to an appropriate final volume of full media. A_2A_R-expressing HEK293T cells were used 48 h post-transfection.

### Preparation of styrene maleic acid co-polymer

Poly(styrene-co-maleic anhydride) with a ratio of 2:1 styrene to maleic anhydride was used as described in [15]. Briefly, a 10% solution of SMA co-polymer in NaOH (1 M) was refluxed for 2 h and allowed to cool to room temperature, then dialysed against buffer before use.

### SMA solubilization of A_2A_R

A_2A_R-expressing yeast membranes, at a final concentration of 40 mg/ml (wet weight) were incubated with SMA (2.5% w/v final concentration) for 20 h at 25°C with gentle stirring. Non-solubilized material was sedimented at 100000 ***g*** for 1 h at 4°C, to yield a supernatant containing A_2A_R–SMALP. HEK293T cells transiently transfected with A_2A_R were washed with ice-cold PBS 48 h post-transfection, then scraped into 1 ml/dish of harvesting buffer (20 mM HEPES, 1 mM EGTA, 1 mM magnesium acetate, pH 7.4) containing 2% (w/v) SMA and 5 units/ml of benzonase. Samples were incubated at 37°C for 1 h before centrifugation at 100000 ***g*** for 1 h at 4°C.

### DDM solubilization of A_2A_R

Yeast membranes containing A_2A_R were solubilized with DDM in solubilization buffer [50 mM Tris/HCl, 10% glycerol, 500 mM NaCl, 2.5% (w/v) DDM, 0.5% (w/v) cholesteryl hemi-succinate, plus complete EDTA-free protease inhibitor cocktail (Roche), pH 8.0] with a final concentration of 40 mg/ml (wet weight). After incubation with slow rotation at 4°C for 2 h, the sample was centrifuged at 100000 ***g*** for 1 h at 4°C to remove the non-solubilized material. For HEK293T cells, a crude membrane preparation was prepared as described previously [20] and the protein concentration determined using the BCA protein assay kit (Pierce) using BSA as standard. A_2A_R-expressing membranes (final protein concentration 0.5 mg/ml) were incubated with solubilization buffer (20 mM HEPES, 1mM EGTA,1 mM magnesium acetate, 2.5% (w/v) DDM, 0.5% (w/v) cholesteryl hemi-succinate, plus complete EDTA-free protease inhibitor cocktail (Roche) for 3 h at 4°C.

### Ni^2+^–NTA affinity purification

The SMA-solubilized preparation was recirculated through a HisTrap HP 1 ml Ni^2+^–NTA (nitrilotriacetate) Sepharose (GE Healthcare) overnight at 4°C. The column was washed with 20 column volumes (cv) of 50 mM Tris/HCl, pH 8.0, 500 mM NaCl, 10% glycerol, 20–60 mM imidazole, complete EDTA-free protease inhibitor. Elution of the A_2A_R–SMALP was achieved using 250 mM imidazole (10 cv) collected in 0.5 ml fractions. Eluted fractions containing A_2A_R–SMALP were pooled, dialysed overnight against buffer (50 mM Tris/HCl, 150 mM NaCl, 10% glycerol, complete protease inhibitor then concentrated using a centrifugal concentrator (Vivaspin 20, 30 kDa cut-off, Sigma)). Western blotting analysis used a primary anti-histidine antibody (Takara Bio Europe) and an anti-mouse-HRP (horseradish peroxidase; NEB) secondary antibody (both at 1:5000 dilution) visualized using a chemiluminescence detection kit (Geneflow).

### Radioligand-binding assays

Competition radioligand-binding assays with A_2A_R–SMALP used [^3^H]ZM241385 (1 nM) as tracer ligand plus competing ligand at the concentrations indicated in a final volume of 250 μl. Non-specific binding was defined by unlabelled ZM241385 (1 μM). After incubation at 30°C for 30 min to establish equilibrium, bound and free ligand were separated by P30 mini-spin gel filtration columns (1000 ***g***, 4 min). Bound ligand in the void volume was quantified by liquid scintillation counting using a Packard Tri-Carb Liquid Scintillation Counter with HiSafe3 (Perkin–Elmer) as cocktail. Binding data were analysed by non-linear regression to fit theoretical Langmuir binding isotherms (with Hill slopes constrained to unity) to the experimental data using PRISM (Graphpad Software Inc.). *K*_i_ values were calculated from IC_50_ values using the Cheng–Prusoff equation to correct for radioligand occupancy [21].

### Gel filtration analysis

Gel filtration experiments were performed at 20°C using a Superdex 200 10/300 GL column pre-equilibrated with buffer (50 mM Tris, 300 mM NaCl, pH 8.0) on an ÄKTA Purifier system (GE Healthcare) with a flow rate of 1 ml/min. All experiments were performed using 50 mM Tris, pH 8, with 300 mM NaCl using a flow rate of 1 ml/min. The column was calibrated using the standards ovalbumin (43 kDa), canalbumin (75 kDa), aldolase (158 kDa), ferritin (440 kDa) and thyroglobulin (669 kDa) (GE Healthcare).

### CD

Data between 260 and 195 nm were collected using a JASCO J-715 Spectrapolarimeter with a 1 mm path length cuvette containing 0.05 mg/ml protein. Each spectrum was collected with a data pitch of 0.5 nm and represents the average of eight scans. Data were also collected using the same parameters for cuvettes containing the relevant buffer to allow subtraction of the buffer contribution.

### Sedimentation velocity analytical ultracentrifugation

Purified A_2A_R–SMALP equilibrated in 10 mM sodium phosphate, pH 8.0, was characterized by analytical ultracentrifugation (AUC). Velocity experiments were performed using a Beckman Coulter XL-I analytical ultracentrifuge (Beckman 693 Coulter) with a Ti50 rotor at 40000 rpm (129000 ***g***) at 4°C. The protein within the cell was monitored by absorbance at 280 nm. Data were then analysed using the c(S) and c(M) routines implemented within SEDFIT, a sedimentation data-fitting program [22]. Parameters for A_2A_R vbar and solvent density and viscosity were calculated using SEDNTERP, a sedimentation interpretation program [23].

## RESULTS AND DISCUSSION

### Detergent-free purification of the A_2A_R expressed in yeast using SMALPs

**Figure 1:**
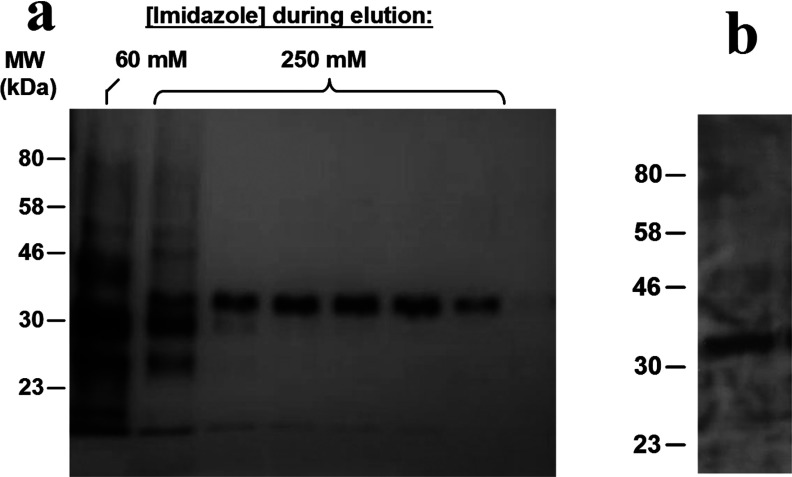
Purification of SMALP-solubilized His-tagged A_2A_R from *P. pastoris* (**a**) The A_2A_R eluted from the Ni^2+^–NTA linked agarose as a single band in silver-stained fractions with 250 mM imidazole. (**b**) Western blot of the 250 mM imidazole fraction with an anti-histidine antibody.

The yeast *P. pastoris* has been utilized extensively to overexpress a wide range of membrane proteins from a variety of organisms, including GPCRs for crystallization [24,25]. A histidine-tagged human A_2A_R was expressed in a genetically-engineered strain of *P. pastoris*, developed specifically for high-level expression of recombinant membrane protein [26], using culture conditions optimized for production of functional receptor. A_2A_R-expressing yeast cells were disrupted [16], then directly solubilized with 2.5% (w/v) SMA co-polymer in the total absence of detergent. The membrane pellet visibly clarified upon exposure to SMA due to SMALP formation. Following removal of non-solubilized material by centrifugation (100000 ***g***, 1h), the A_2A_R–SMALP was purified using Ni^2+^–NTA linked agarose (Figure 1a). The A_2A_R eluted as a single band in fractions containing 250 mM imidazole. The identity of the band as the A_2A_R was confirmed by Western blotting using anti-histidine antibody to detect the histidine-tagged A_2A_R (Figure 1b) and partial sequencing of the purified protein using Fourier-transform ion cyclotron resonance (FTICR) MS. The FTICR identified three peptides YNGLVTGTR, QMESQPLPGER and SHVLRQQEPFK corresponding to A_2A_R residues 112–120 (part of intracellular loop 2), 210–220 (part of intracellular loop 3) and 305–315 (part of the C-terminal tail) respectively.

Radioligand-binding assays with [^3^H]ZM241385 established that the binding (*B*_max_) of the purified A_2A_R–SMALP preparation was 20100 pmol/mg protein, consistent with the theoretical value for fully active receptor (21300 pmol/mg protein), compared with 9.5 pmol/mg protein in the original yeast membrane. Competition ligand binding assays using a range of ligands with A_2A_R–SMALP were consistent with established A_2A_R pharmacology (International Union of Pharmacology (IUPHAR) database) and comparable to A_2A_R binding in the original yeast membranes (Table 1).

**Table 1:** Pharmacological characterization of A_2A_R–SMALP from *P. pastoris* For each ligand, the p*K*_i_ value is presented for binding to A_2A_R expressed in *P. pastoris* membranes and also A_2A_R–SMALP generated from the *P. pastoris* membranes. Data are mean ± S.E.M. (*n*=3).

Ligand	Yeast membrane	Yeast-SMALP
ZM241385	7.95±0.45	7.79±0.14
XAC	6.53±0.24	7.16±0.18
NECA	5.66±0.26	5.43±0.1
Theophylline	3.82±0.30	4.13±0.1

Several nanoscale self-assembly reagent systems have been reported for purifying membrane proteins including bicelles, amphipols, scaffolding protein-stabilized nanodiscs and SMALPs [27]. However, of these, only SMALP-solubilization of GPCRs; (i) avoids exposure to detergent at any stage, (ii) preserves the native composition of the annular lipid in close association with the receptor and (iii) retains the lateral pressure exerted within the membrane bilayer that has been shown to be important for maintaining the conformation of membrane proteins [28]. Previous studies have shown that membrane proteins could be SMALP-solubilized from prokaryotes, such as archaea and bacteria [15,29] and also from eukaryotic cells [30], but this is the first report that a GPCR can be SMALP-solubilized and furthermore that the active receptor can be purified to homogeneity as a GPCR–SMALP without the use of detergent at any stage.

### Increased thermostability of A_2A_R–SMALP compared with detergent-solubilized A_2A_R

**Figure 2:**
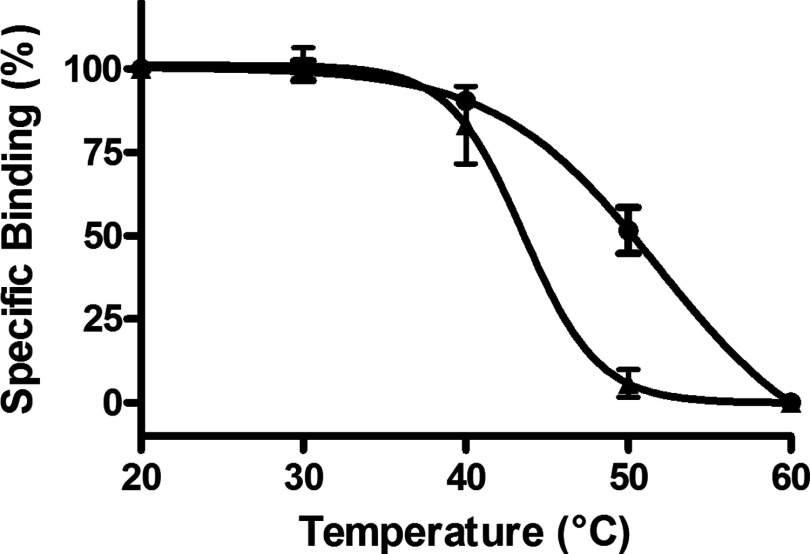
Thermostability of A_2A_R–SMALP and DDM-solubilized A_2A_R from *P. pastoris* A_2A_R–SMALP (●) and DDM-solubilized A_2A_R (▲) prepared from *P. pastoris* overexpressing A_2a_R were incubated for 30 min at the stated temperatures, chilled on ice, before specific binding of [^3^H]ZM241385 was determined as described in ‘Methods’. Data are expressed as specific binding relative to the 20°C data point (mean ± S.E.M. of three separate experiments performed in triplicate).

An acknowledged common problem with detergent solubilization of GPCRs is destabilization of the receptor in the detergent micelle compared with the native plasma membrane. As the A_2A_R–SMALP preserves the annular lipid in close contact with the receptor in the membrane, it was reasoned that A_2A_R would be more thermostable in a SMALP than when detergent-solubilized by DDM, a widely employed detergent for GPCR solubilization. The antagonist [^3^H]ZM241385 is widely used to quantify functional A_2A_R-binding capability using radioligand-binding assays. A_2A_R–SMALP and DDM-solubilized A_2A_R from *P. pastoris* membranes were incubated in parallel at various temperatures and then the residual specific binding of [^3^H]ZM241385 was determined. From data presented in Figure 2, it can be seen that the SMALP conferred a marked increase in A_2A_R thermostability of ∼5.5°C over the detergent micelle, with the T_50_ value increasing from T_50_=44.4±0.27°C (*n*=3) for A_2A_R–DDM to T_50_=49.9±1.19°C (*n*=3) for A_2A_R–SMALP.

### Biophysical characterization of purified A_2a_R–SMALP

**Figure 3:**
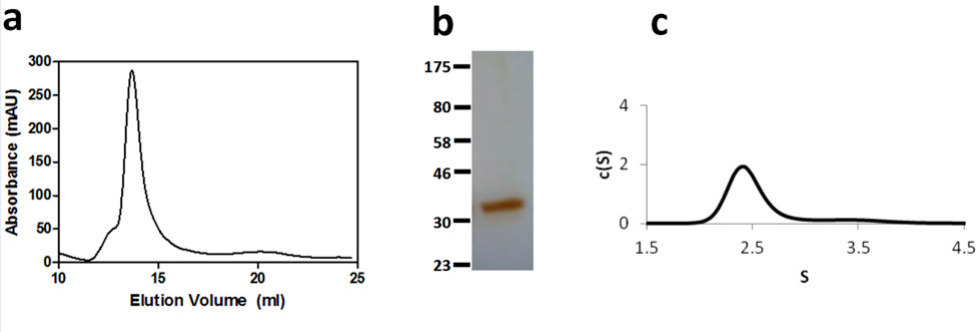
Analysis of A_2A_R–SMALP from *P. pastoris* by size-exclusion chromatography and AUC (**a**) elution profile of A_2A_R–SMALP from a Superdex 200 10/300GL size exclusion column with absorbance measured at 280 nm. (**b**) silver stained SDS/PAGE of the main peak eluted from the size exclusion chromatography. (**c**) plot of c(S) compared with sedimentation coefficient for A_2A_R–SMALP.

Characterization of the A_2A_R–SMALP by size-exclusion chromatography revealed a mono-dispersed particle (Figure 3a). Analysis of the main peak by SDS/PAGE revealed a single protein band (Figure 3b) with a migration consistent with the A_2A_R (compare with Figure 1). AUC of the A_2A_R–SMALP confirmed the presence of a single major particle species with a sedimentation coefficient of 2.3S (Figure 3c).

**Figure 4:**
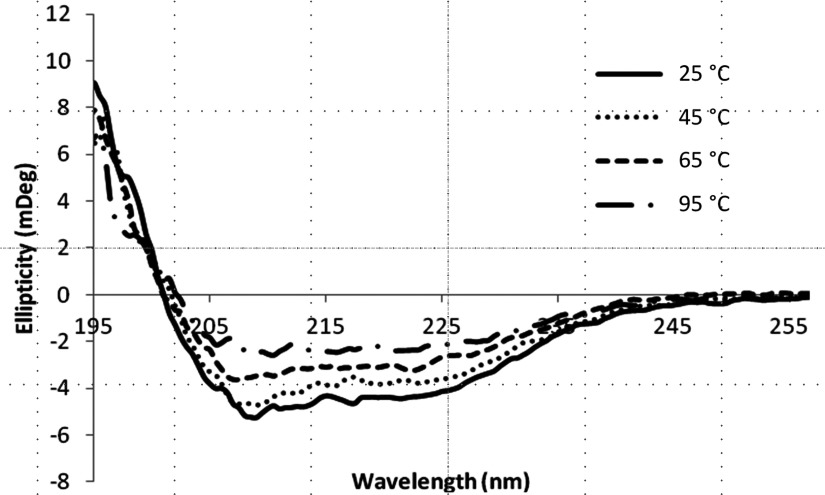
The effect of temperature on A_2a_R structure revealed by CD spectra of purified A_2a_R–SMALP CD spectra were collected using a 1-mm path length cuvette and averaged over eight scans in the far-UV region (195–260 nm). Spectra were corrected for the buffer signal.

The vast majority of membrane proteins stabilized in nanodiscs to-date have used the MSP-based system developed by Sligar and colleagues [12]. In this MSP method, the lipid disc is stabilized by an annulus of scaffolding proteins which can interfere with spectrophotometric studies on the embedded membrane protein of interest. In contrast, the lipid disc in our SMALP system is stabilized by a non-proteinacious polymer, which we have already shown in a previous study, does not suffer from the same limitations [15]. This allowed the conformation of the SMALP-encapsulated A_2A_R to be studied using CD spectroscopy. It is known from solved crystal structures that the A_2A_R possesses a high α-helix content due to the seven transmembrane helical bundle that is a conserved feature of GPCR architecture [31,32]. The far-UV (195–260 nm) CD spectrum of purified A_2A_R–SMALP showed negative minima at 208 and 222 nm consistent with a folded protein containing a high degree of α-helix (Figure 4), consistent with the known secondary structure content of the A_2A_R. In contrast, an unfolded protein would have a negative minimum at 200 nm. CD was also used to assess the thermal stability of the encapsulated A_2A_R. These data show that as the temperature increases from 25°C to 95°C the intensity of the 208 and 222 nm features reduces until at 95°C the intensity is less than 50% of that observed at low temperature. This indicates that the protein is steadily losing secondary structure as the temperature increases. Closer examination of the CD spectra shows a single isodichroic point at ∼201 nm. This indicates that the thermal denaturation process is a two state process most probably correlating with a folded to unfolded transition. Comparison of the CD spectra in Figure 4 to the thermostability binding data (Figure 2) revealed that the change in the CD spectrum observed between 25°C and 65°C reflected the structural changes underlying the complete loss of [^3^H]ZM241385 binding by the A_2A_R. The A_2A_R was not fully denatured when the radioligand-binding capability was lost; however, as α-helix content was still apparent in the CD spectrum at 65°C. The proportion of α-helix decreased further as the temperature was raised from 65°C to 95°C but even at 95°C the A_2A_R CD signal had not completely changed to that of a random coil (characterized by an intense negative signal at 200 nm and a positive signal at 218 nm [33]). This indicates that the helical secondary structure of the A_2A_R is resistant to thermal denaturation in SMALPs. Retention of α-helical content has also been reported for rhodopsin during denaturation studies with combinations of different denaturants. Furthermore, it was concluded that surface elements within the rhodopsin structure were susceptible to denaturation, becoming more flexible [34], whereas a cluster of interconnected segments from multiple transmembrane helices preserved a rigid core [35]. This could explain our observations on the effect of increasing temperature on the A_2A_R structure and function. Molecular dynamics simulations indicate that the process of a ligand binding to its cognate GPCR progresses through an intermediate state in which the ligand binds initially to an extracellular vestibule prior to docking in the classical ‘orthosteric’-binding site [36,37]. The rhodopsin unfolding studies cited above indicate that surface elements within the A_2A_R architecture would be more susceptible to structural perturbation than the relatively rigid α-helical core. This suggests a feasible mechanism for our observations that the ligand-binding capability of the A_2A_R was lost at temperatures which preserved significant α-helical content, albeit lower than native A_2A_R. If the conformations of extracellular structural elements that contribute to the approach of a ligand to the orthosteric-binding site via the extracellular vestibule were susceptible to thermal disruption, then ligand binding capability would be ablated but core α-helical structure would be largely retained.

### Characterization of A_2A_R–SMALP from transfected HEK293T cells

The utility of SMALPs for purifying a functional human GPCR from *P. pastoris* for investigation by biophysical approaches has been demonstrated above. However, the exact lipid composition of the yeast plasma membrane is different from mammalian cells, most notably perhaps in lacking cholesterol and possessing ergosterol [38]. The nature of the lipids juxtaposed to a GPCR can affect the function. For example, cholesterol can modulate the activity of some GPCRs [39,40], can affect receptor conformation [41] and has been co-crystallized with GPCRs, which led to a ‘cholesterol consensus motif’ being proposed as part of a specific cholesterol-binding site within the architecture of some GPCRs [42]. As the SMALP-solubilization process involves the SMA polymer excising the receptor embedded in a nanodisc of native membrane bilayer [15] the lipid composition preserved in the A_2A_R–SMALP is dictated by the composition of the cell membrane in which the receptor was expressed. There will be instances, such as drug discovery assays, when it will be desirable to preserve the more physiological annular lipid composition provided by a mammalian cell membrane. Consequently, we also characterized A_2A_R–SMALP from transfected HEK293T cells.

**Figure 5:**
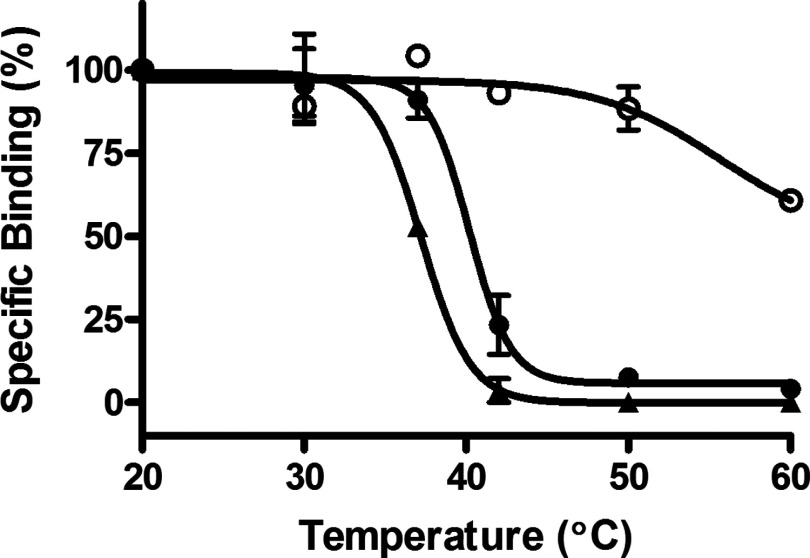
Thermostability of A_2a_R–SMALP, DDM-solubilized A_2a_R and membrane-bound A_2a_R prepared from HEK293T cells A_2a_R–SMALP (●), DDM-solubilized A_2a_R (▲) or membranes (○), prepared from HEK293T cells transfected with A_2a_R, were incubated for 30 min at the stated temperatures, then chilled on ice, before specific binding of [^3^H]ZM241385 was determined as described in ‘Methods’. Data are expressed as specific binding relative to the 20°C data point (mean ± S.E.M. of three separate experiments performed in triplicate).

HEK293T cells, expressing A_2A_R, were directly solubilized with 2.0% (w/v) SMA, in the total absence of detergent, to generate a functional A_2A_R–SMALP preparation of 2.0±0.24 pmol/mg protein (*n*=3), equivalent to 23.3±2.75% (*n*=3) recovery of A_2A_R. Characterization of this A_2A_R–SMALP by competition radioligand-binding using a range of ligands with [^3^H]ZM241385 as tracer confirmed retention of typical A_2A_R pharmacology (Table 2). The A_2A_R–SMALP from HEK293T cells exhibited an increase in thermostability of 4°C compared with the corresponding detergent (DDM)-solubilized A_2A_R (Figure 5), with the T_50_ value increasing from T_50_=36.2±0.52°C (*n*=3) for A_2A_R–DDM to T_50_=40.2±0.44°C (*n*=3) for A_2A_R–SMALP. The A_2A_R–SMALP was not as stable as A_2A_R embedded in the native HEK293T cell membrane however (Figure 5).

**Table 2:** Pharmacological characterization of A_2A_R–SMALP from HEK293T cells For each ligand, the p*K*_i_ value is presented for binding to A_2A_R in HEK293T membranes and also A_2A_R–SMALP generated from the HEK293T cell membranes. Data are mean ± S.E.M. (*n*=3).

Ligand	HEK membranes	HEK–SMALP
ZM241385	8.87±0.1	8.53±0.04
XAC	7.17±0.19	6.38±0.16
NECA	5.29±0.1	5.39±0.41
Theophylline	4.87±0.12	4.97±0.27

**Figure 6:**
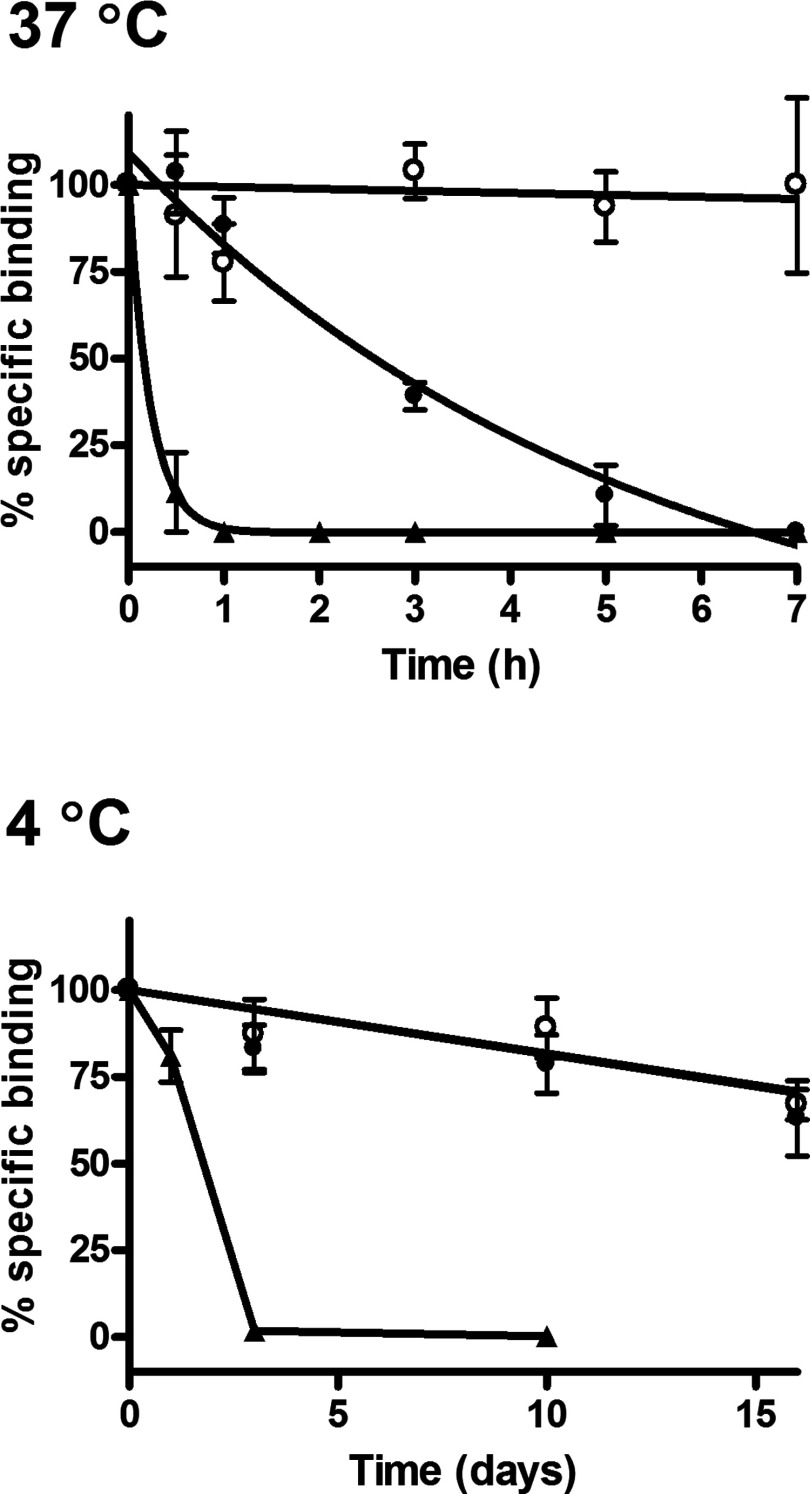
Comparison of A_2a_R stability in different preparations at 37C and 4C A_2a_R–SMALP (●), DDM-solubilized A_2a_R (▲) or membranes (○), prepared from HEK293T cells transfected with A_2a_R, were incubated at 37°C (top panel) or 4°C (bottom panel), for the stated times. Specific binding of [^3^H]ZM241385 was determined at each time point. Data are expressed as specific binding relative to time zero (mean ± S.E.M. of three separate experiments performed in triplicate).

**Figure 7:**
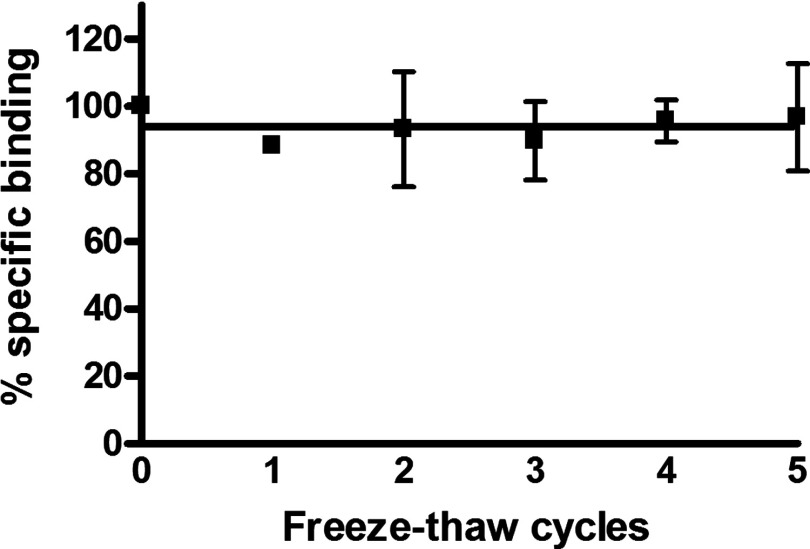
Effect of repeated freeze-thaw cycles on A_2a_R–SMALP binding capability A_2a_R–SMALP was subjected to repeated freeze/thaw cycles and specific binding of [^3^H]ZM241385 determined after each cycle. Data are expressed as specific binding relative to binding before freezing (mean ± S.E.M. of three separate experiments performed in triplicate).

Given the potential utility of the GPCR–SMALP for receptor-based *in vitro* assays and screens, the stability of the A_2A_R–SMALP at the physiological temperature of 37°C and the storage temperature of 4°C was investigated together with the resistance to repeated freeze-thaw cycles. A comparison of the loss of ligand binding capability of the A_2A_R at 37°C with time is presented in Figure 6 for A_2A_R–DDM, A_2A_R–SMALP and cell membranes. The improved stability of the A_2A_R–SMALP over A_2A_R–DDM at 37°C is very marked, with a 7-fold increase in the half-life of [^3^H]ZM241385 binding (A_2A_R–DDM, t_½_=21±7 min; A_2A_R–SMALP, t_½_=148±13 min). This improvement is particularly apparent after 1 h, with no specific binding detected for the detergent-solubilized receptor, whereas 85±5% of the binding was retained in the A_2A_R–SMALP (Figure 6). Although receptor stability was increased in the SMALP, it did not match receptor stability in the native HEK293T membranes at 37°C, suggesting that not all the stabilizing factors of the plasma membrane are incorporated into SMALPs. In contrast, the stability of A_2A_R–SMALP at 4°C was essentially indistinguishable from that of membranes with t_½_ ≥16 days, whereas A_2A_R–DDM exhibited a t_½_=1.8±0.3 days (mean ± S.E.M.). The resilience of A_2A_R–SMALP was also evident from repeated freeze/thaw cycles (Figure 7). Even after five freeze/thaw cycles on the same sample, there was no decrease in [^3^H]ZM241385 binding capability of the A_2A_R–SMALP. In contrast, specific binding to the A_2A_R–DDM preparation was completely lost after a single freeze-thaw cycle. Our data indicate that it is feasible to SMALP solubilize GPCRs and store them at 4°C until required. Interestingly, the increase in thermostability endowed on A_2a_R by the SMALP compared with DDM was approximately 5°C for both yeast and HEK 293T, suggesting that the increased thermostability is an inherent property of the SMALP rather than due to differences in the lipid composition of the original membrane.

## CONCLUSION

Strategies to ameliorate the detrimental effect of exposing GPCRs to detergents have included; modifying the detergent to reduce perturbation to the receptor [4], modifying the receptor to increase stability to detergent either by alanine scanning [43] or by molecular evolution [44] and simply reducing the detergent exposure time by inserting the detergent-solubilized receptor into nanodiscs stabilized by scaffolding proteins [9]. However, the ideal option would be to extract GPCR from the membrane, for purification or downstream assays, without the requirement for detergent. In the present study, we report the first purification of a GPCR (human A_2a_R), without exposure to detergent at any stage, by exploiting spontaneous encapsulation direct from a membrane into a nanoscale SMALP. The A_2A_R–SMALP, generated from both yeast and mammalian cells, exhibited increased thermostability compared with DDM-solubilized receptor that will facilitate use in receptor-based assays/screens. Furthermore, in contrast with nanodiscs stabilized by scaffold proteins, the non-proteinaceous nature of the SMA polymer does not interfere with biophysical characterization of the embedded receptor.
